# The Influence of Seasonal Variations in Clinical Trials Based on Gut Microbiota Studies

**DOI:** 10.3390/microorganisms13102386

**Published:** 2025-10-17

**Authors:** Giovanna Cocomazzi, Concetta Panebianco, Annamaria Vallelunga, Daniele De Ruvo, Lino Del Pup, Serena Smeazzetto, Monica Antinori, Valeria Chimienti, Gabriele Maggio, Concetta Finocchiaro, Viviana Contu, Valerio Pazienza

**Affiliations:** 1 Gastroenterology Unit, Fondazione IRCCS “Casa Sollievo della Sofferenza” Hospital, Viale dei Cappuccini 1, 71013 San Giovanni Rotondo, Italy; g.cocomazzi@operapadrepio.it (G.C.); panebianco.c@gmail.com (C.P.); 2 Studio Vallelunga-Functional Nutrition, Via Manara 11, 04019 Terracina, Italy; vallelungaannamaria@gmail.com; 3 Gynaecology, Obstetrics and Reproductive Medicine Affidea Promea, Via Menabrea 14, 10126 Torino, Italy; daniele.deruvo@gmail.com; 4 Gynecological Endocrinology and Fertility, University Sanitary Agency Friuli Central (ASUFC), Via San Valentino 18, 33100 Udine, Italy; info@delpupginecologia.it; 5 NEXT Genomics Srl, 50019 Sesto Fiorentino, Italy; serena@personalnext.it; 6 Raprui Clinic, Via Timavo, 2, 00195 Roma, Italy; monica.antinori@raprui.com; 7 VMC-OsteoKinetic Studio, Via Roma, 26/a, 20096 Pioltello, Italy; v.chimienti.osteopata@hotmail.com; 8 Pia Fondazione Cardinale Giovanni Panico, Via S. Pio X, 4, 73039 Tricase, Italy; gabrielemaggio.gm@alice.it; 9 Integrative Medicine Unit, Humanitas Gradenigo, Corso Regina Margherita 8/10, 10153 Torino, Italy; ettafinocchiaro@gmail.com (C.F.); viviana.contu@gradenigo.it (V.C.)

**Keywords:** gut microbiota, seasonality, dietary, non-communicable chronic diseases

## Abstract

Seasonality is a key determinant in shaping the composition and function of the human gut microbiota, exerting its influence through multiple interconnected factors. These include seasonal variations in diet, environmental conditions (such as temperature, humidity, and sunlight exposure), behavioral patterns (physical activity, time spent outdoors), and the incidence of seasonal infections. These changes are most visible in certain populations where food availability follows a seasonal pattern. Increasing evidence suggests that gut microbiota composition undergoes seasonal variations, which may have significant implications for human health. In patients with non-communicable chronic diseases (NCDs), where microbiota dysbiosis plays a crucial role in disease progression, understanding the seasonal effects on gut microbiota is essential. These dynamic changes impact the gut microbiota immune system interaction and may contribute to the onset or exacerbation of various diseases, including chronic inflammatory, metabolic, and autoimmune disorders. Most clinical trials on probiotics do not consider seasonality as a confounding variable, which could impact the interpretation of results and therapeutic efficacy, potentially biasing estimates of intervention efficacy or associations with disease. This review examines the current evidence on gut microbiota seasonality, discusses its potential bias, and outlines methodological considerations for future clinical trials.

## 1. Introduction

In recent years, scientific interest in the gut microbiota has grown exponentially, revealing the central role this microbial ecosystem plays in maintaining homeostasis and modulating numerous pathophysiological processes [1]. The microbiota consists of approximately one hundred trillion microbial cells that colonize our bodies. Ninety-five percent of the bacteria that make up the complete human microbiota reside in the gastrointestinal system, with the remaining fraction distributed across other organs’ mucosal and epithelial surfaces [2], and they play a significant function in human physiology, influencing metabolic processes, immune modulation, nutrient absorption, and the host’s resistance to pathogens [3]. Alterations in the composition or function of this microbial ecosystem, commonly referred to as dysbiosis, have been implicated in metabolic and immune system dysfunction, in the development and progression of various non-communicable chronic diseases (NCDs), including obesity, type 2 diabetes, cardiovascular disease, cancer, and inflammatory bowel disease [4]. The human gut microbiota differs substantially among individuals because of a number of factors such as host genetic background, diet, cultural practices, socioeconomic level, exposure to xenobiotics, infections and lifestyle (alcohol, smoking, physical activity) [5] but an important dimension often overlooked is the seasonality. Seasonal variations represent a still underestimated but potentially crucial temporal dimension, especially in the context of clinical research and understanding of NCDs. Seasonal fluctuations and behavioral factors such as air temperature, diet composition, physical activity, sun exposure and the prevalence of infections are capable of inducing shifts in microbiota structure and function [6,7]. Studies in traditional populations, such as the Hadza in Tanzania, have documented significant seasonal changes in gut microbiota composition, suggesting adaptive plasticity with respect to diet and environment [6]. Even in Western populations, where seasonality is less pronounced nutritionally, emerging evidence indicates that the changing seasons can induce measurable variations in the microbiome, affecting the relative abundance of key taxa and the functional expression of microbial genes [7,8]. This seasonal variability introduces a potential source of bias in clinical trials studying the microbiota; in many studies, in fact, the time of collection is neglected or not reported, compromising data reproducibility and interpretation [9]. In addition, the interaction between the gut microbiota and NCDs, including obesity, diabetes, cardiovascular disease and neuropsychiatric disorders, can be mediated by seasonal environmental factors that, if not taken into account, lead to concealing or incorrectly exaggerating observed associations [10]. It is therefore essential to understand how seasonality modulates gut microbiota trajectories to improve clinical trial design and interpretation of results from a precision medicine perspective. In this review, current evidence regarding the seasonality of the gut microbiota will be explored, analyzing the role of environmental, dietary, and behavioral factors drivers of these temporal variations. The methodological biases that such fluctuations may introduce into clinical trials will also be discussed, and finally, the implications of these seasonal dynamics in the pathogenesis and management of implications for chronic disease dynamics will be examined. We also address how these changes may introduce bias in microbiota research and propose methodological strategies such as season-stratified randomization and adaptive trial design to mitigate this risk. Integrating the temporal dimension of seasonality into microbiota studies is not only critical for scientific rigor, but also for the advancement of precision medicine strategies.

## 2. Search Strategies

To select relevant studies, the literature discussed in this review was identified through a bibliographic search on PubMed, Scopus, and Web of Science covering the period from January 2000 to September 2025 using the following keywords: gut microbiota, seasonality, impact of seasonal variations, diet, and chronic non-communicable diseases. Boolean operators (AND, OR) were used to refine the search: Gut microbiota AND seasonality; Diet OR Food Available AND Seasonality; Environmental AND Behavioral Factors AND Seasonality; Sunlight OR Vitamin D AND Seasonality; Seasonality AND Physical Activity impact on Gut Microbiota; Thermogenic OR Climate impact on Gut Microbiota; Seasonal Infections AND Gut Microbiota; Circadian Rhythms AND Gut Microbiota; Seasonality AND Non-Communicable Chronic Diseases; Seasonality Bias AND Methodological strategies. Only articles in English were considered.

## 3. Mechanisms and Evidence of Gut Microbiota Seasonality

### Seasonal Food Availability and Dietary Influence

Diet is one of the most powerful modulators of gut microbiota composition, and its seasonal variability plays a crucial role in driving microbial fluctuations throughout the year. This is particularly evident in traditional or subsistence-based populations, where dietary patterns closely mirror seasonal food availability. Studies on hunter–gatherer populations, such as the Hadza in Tanzania, have shown profound shifts in gut microbiota composition between the wet and dry seasons. During the dry season, when fiber-rich tubers are the primary food source, the gut microbiota is enriched in taxa associated with complex carbohydrate fermentation. In contrast, during the wet season, increased consumption of animal-derived foods and honey leads to shifts favoring protein- and sugar-metabolizing bacteria [6]. Even in industrialized societies, where food availability is less dependent on seasonal cycles, fluctuations in food consumption patterns persist. In temperate regions, winter diets tend to be higher in processed foods and animal products, while summer diets often include more fresh fruits, vegetables, and fiber. These dietary changes can lead to fluctuations in microbial diversity and metabolic outputs, potentially affecting host immune function and inflammatory status [7]. In the Hutterite population of North Dakota, characterized by communal living and highly synchronized diets, seasonal differences in gut microbiota composition were observed despite minimal inter-individual dietary variability. Specifically, in the summer season when fiber and fresh fruits are consumed, the gut microbiota is rich in Bacteroidetes and depleted in Actinobacteria and Firmicutes compared to winter [7]. Similar patterns were reported in Mongolians populations, where traditional winter diets rich in meat and dairy lead to distinct gut microbiota profiles, possibly reflecting microbial adaptation to high-fat, high-protein diets during colder months [11]. Consuming meats and dairy products during the winter, alters the microbial makeup and it has an impact on health and metabolism [12]. It is possible that Mongolians’ microbiota has adjusted to these seasonal variations by exhibiting exceptional flexibility and reacting quickly to dietary modifications. Researchers examined the gut microbiota composition of 769 participants in a study focused on seasonal variations in the makeup of the human gut microbiota in the Ukrainian population [13]. The results showed that the relative abundance of the gut microbiota’s major taxonomic groups varied depending on the month of sampling. Actinobacteria were more prevalent and Bacteroidetes were less abundant in summer samples than in other seasons, although Firmicutes concentration did not vary seasonally. The Firmicutes/Bacteroidetes (F/B) ratio was substantially greater in samples collected during the summer versus the winter ones, implying that seasonal sampling may influence the outcomes of human microbiome research, potentially introducing bias into the estimations. Animal studies further reinforce the role of seasonal dietary changes in shaping the gut microbiota. A study on the impact of seasonal diet on the gut microbiota of 302 yaks showed that different bacterial taxa dominate in summer versus winter, reflecting shifts in forage availability. During the warm season, when diets are higher in fiber and lower in protein, the strain Ruminococcaceae_UCG-005 was more common, whereas during the cold season, *Akkermansia* and *Eubacterium* WCHB1-41 were more common [14]. *Akkermansia*, in particular, has the ability to digest mucins and convert them to monosaccharides or amino acids, supplying nourishment to other gut bacteria [15]. In hibernating animals, seasonal variations show even more dramatic seasonal remodeling of the gut microbiota. In ground squirrels, hibernation is associated with a decline in Firmicutes which rely on exogenous substrates (food-derivated) and an increase in Bacteroidetes and Verrucomicrobia, which can metabolize host-derived compounds such as mucins [16]. This microbial shift is accompanied by a reduction in butyrate production, a short-chain fatty acid (SCFA), vital for maintaining intestinal barrier function, epithelial integrity, controls apoptosis and mucus [17,18,19]. These findings suggest that seasonal changes in microbial metabolism may contribute to mucosal atrophy observed during periods of prolonged fasting or hibernation. Taken together, this body of evidence underscores the gut microbiota’s responsiveness to seasonal dietary shifts across a broad spectrum of environments and species. These dynamic fluctuations in microbial community structure and metabolic output may, in turn, influence host physiology and disease risk, particularly in susceptible individuals.

## 4. Environmental and Behavioral Factors

**Figure 1:**
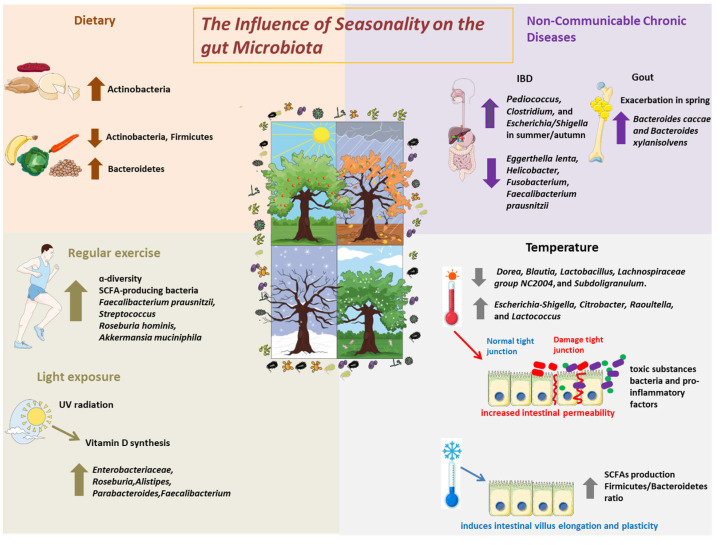
The Influence of Seasonality on the Gut Microbiota. Schematic representation of the main seasonal environmental and behavioral factors that influence gut microbiota composition and function. Environmental factors include temperature variation, sunlight exposure, and seasonal infections, while behavioral factors encompass changes in physical activity and dietary intake. These elements interact with the host immune system, circadian rhythm, and metabolic pathways, collectively shaping microbial diversity and function across seasons.

Beyond dietary influences, seasonality exerts a multifactorial influence on the gut microbiota through a combination of environmental exposures (e.g., sunlight, temperature, infections) and behavioral changes (e.g., physical activity, sleep, circadian rhythms), as represented in Figure 1. These factors, although distinct, are interconnected and modulate the host–microbiota interaction through immune and metabolic pathways.

### 4.1. Effect of Sunlight Exposure and Vitamin D Production on Gut Microbiota

Seasonal variation in sunlight exposure directly impacts endogenous vitamin D synthesis, which in turn influences immune system function and gut microbial composition. Studies suggest that vitamin D deficiency, more common in winter, is linked with reduced microbial diversity and increase in pro-inflammatory species [20]. In inflammatory bowel disease (IBD) patients, seasonal variation in vitamin D levels has been linked to changes in microbiota composition [21]: higher relative abundances of *Pediococcus*, *Clostridium*, and *Escherichia/Shigella* were observed during summer/autumn, while *Eggerthella lenta*, *Helicobacter*, *Fusobacterium*, and *Faecalibacterium prausnitzii* decreased in the same period. Vitamin D modulates tight junction protein expression and reduces gut permeability, a critical factor in preventing microbial translocation and systemic inflammation [22]. Supplementation in IBD patients has been shown to restore the abundance of beneficial genera such as *Faecalibacterium*, *Roseburia*, and *Alistipes* [23], highlighting the interplay between sunlight exposure, immune modulation, and microbial ecology. Vitamin D supplementation is associated with changes in the gut microbiota through various mechanisms. Among these, VDR receptors expressed in the intestinal epithelium and in intestinal immune cells play a central role [24]. VDR regulates the transcription of genes involved in immune response, antimicrobial peptide production, and barrier function [25].

In the absence of vitamin D binding to VDR receptors, increased intestinal permeability, inflammation, and alterations in microbial composition are observed. Vitamin D stimulates the production of peptides such as cathelicidin and β-defensins, which help control the microbial population and suppress pathogens [26]. In animal models, vitamin D deficiency leads to a reduction in these antimicrobial peptides [27]. Some metabolites produced by bacteria (e.g., SCFAs, fermentation products) can modulate VDR expression, influence vitamin D metabolism, and modulate the immune system. There may therefore be a bidirectional interaction: vitamin D modifies the composition of the microbiota, but the microbiota can also influence the availability and action of vitamin D [28].

Furthermore, it has been observed that when the intestinal barrier is compromised, lipopolysaccharides (LPS) and other microbial components can pass into the bloodstream and induce systemic inflammation; vitamin D helps mitigate these effects by improving the barrier and immune response [29].

### 4.2. Thermogenic Adaptation and Climate-Related Effects on Gut Microbiota

Environmental temperature influences not only human behavior but also microbial ecology through indirect pathways. For example, the rising of the temperature also impacts the growth of organisms in the soil, since the loss of soil labile carbon promotes the growth of oligotrophic bacteria (which can survive in nutrient-poor environment) over copiotrophic bacteria (which need nutrient-rich environment to thrive). This loss in soil biodiversity is reflected in the micronutrient content of crops and, ultimately, in the human gut bacteria, which may lack fundamental compounds for their metabolism [30]. Moreover, it has been shown that the direct content with the soil modulates gut microbiota, as shown by a higher Bacteroidetes/Firmicutes ratio and improved immunity in mice [31].

Increased body temperature in response to environmental heat or in situations of stress and inflammation can also promote increased intestinal permeability by modifying the structure of tight junctions, promoting the entry of toxic substances and bacteria [32]. Heat stress results in oxidative stress and increases intestinal permeability promoting inflammation and multiorgan lesions, which in turn causes a decrease in the percentage of bacterial species like *Dorea*, *Blautia*, *Lactobacillus*, and *Subdoligranulum* and a greater abundance of pathogenic bacteria like *Escherichia-Shigella*, [33]. Additionally, during the colder months, the consumption of high-fat diets exacerbates this permeability and facilitates low-grade inflammation, contributing to metabolic diseases such as type 2 diabetes [34]. Seasonal exposure to cold environments also triggers specific microbial adaptations. Recent evidence demonstrates that the gut microbiota plays a pivotal role in energy homeostasis during cold exposure [35], showing that cold-induced shifts in microbial composition increase the F/B ratio, termed the “cold microbiota”, promote adaptive thermogenesis, enhance insulin sensitivity, and reduce adiposity when transferred to germ-free mice. Specifically, this microbial configuration supports browning of white adipose tissue and activates thermogenic pathways in brown adipose tissue. In parallel, it induces intestinal remodeling, including villus elongation and increased absorptive capacity, thereby compensating for the elevated energy demand. These effects are mediated in part by increased production of SCFAs, which activate FFAR2/3 receptors and downstream signaling cascades. The depletion of *Akkermansia muciniphila*, observed in the “cold microbiota”, appears to facilitate this intestinal plasticity during cold adaptation, suggesting that microbiota-mediated responses to environmental stressors may be metabolically advantageous in specific contexts [35].

### 4.3. Effect of Seasonal Infections and Immune System Interactions on Gut Microbiota

The gut microbiota plays a central role in regulating the immune system through a bidirectional communication. It shapes immune development and function via microbial metabolites—such as SCFAs, tryptophan metabolites, and microbial-associated molecular patterns (MAMPs)—which help maintain gut barrier integrity, modulate inflammation, and support mucosal immunity [36,37,38]. Seasonal environmental changes particularly during the winter can influence this delicate balance. For example, studies in hibernating mammals such as ground squirrels show that cold-induced microbiota changes lead to increased gut permeability and mucosal atrophy [16,39]. Despite this, an adaptive immune tolerance develops, marked by elevated anti-inflammatory cytokines and secretory IgA, which help contain inflammation during this metabolically constrained state [40]. In humans, winter months are associated with increased incidence of respiratory infections, which indirectly alter the gut microbiota through systemic inflammation, fever and widespread use of antibiotics. Influenza, for instance, has been shown to significantly disrupt gut microbial composition via Th17 cells dependent mechanisms, increasing susceptibility to intestinal inflammation [41]. In particular, after influenza virus infection, the number of *Lactobacillus* decreases, while the *Enterobacteriaceae* increase [41]. Similarly, Li et al. demonstrated that children with recurrent respiratory infections experience changes in their gut microbiota, such as reduced microbial diversity, a decrease in butyrate-producing species, and a lower abundance of *Bifidobacterium*, which is very important in regulating the immune system, as well as an increase in pathogenic microorganisms such as *Enterococcus* [42]. Viral and bacterial infections during seasonal fluctuations also play a role in shaping the gut microbiota. Antibiotic exposure, which often peaks during winter due to upper respiratory infections, is a major driver of microbial dysbiosis [43]. Broad-spectrum antibiotics reduce microbial diversity and deplete key commensals, promoting metabolic changes, the expansion of opportunistic pathogens and increasing intestinal vulnerability to infections like *Clostridium difficile* [44]. A study by Ramirez et al. showed that β-lactam use substantially shifted microbial composition, increasing the Bacteroidetes/Firmicutes ratio and favoring antibiotic-resistant strains [45]. These shifts can have long-term consequences, including metabolic disturbances and immune dysregulation. For instance, seasonal variations have a significant impact on biliary microbiota composition and antibiotic resistance in patients with hepato-pancreatic-biliary cancer [46].

### 4.4. Seasonal-Dependent Physical Activity Impact on Gut Microbiota

Behavioral changes, including physical activity patterns, also follow seasonal trends and have been shown to affect microbiota composition. Regular exercise is associated with increased microbial diversity and higher levels of SCFA-producing bacteria, which confer anti-inflammatory effects and support metabolic health [47]. Exercise has been shown to regulate the gut microbiota through various mechanisms including enhanced intestinal transit, modulation of neurotransmitters and hormones and release of myokines [48]. Furthermore, physical activity improves intestinal barrier function, ensuring proper bowel function; it improves the Bacteroidetes/Firmicutes ratio, an important factor involved in gastrointestinal disorders and weight loss, with a consequent reduction in the incidence of obesity [49]. A study by Bressa et al. reported that active women had a greater abundance of beneficial species such as *Faecalibacterium prausnitzii*, *Roseburia hominis* and *Akkermansia muciniphila* compared to sedentary controls [50]. Similarly, seasonal variation in the gut microbiota has also been observed in Japanese handball players with significantly higher alpha-diversity and higher relative abundance of *Faecalibacterium* and *Streptococcus* (SCFAs-producing genera) compared with nonathletic controls [51]. The SCFAs produced by the bacteria can activate AMPK in the muscle. AMPK regulates the activity of a variety of factors involved in cholesterol homeostasis as well as lipid and glucose metabolism in muscle [52]. Furthermore, SCFAs enhance PYY (a satiety hormone) levels in the plasma via Ffar2/3 receptors in the colon [53]. Related to physical activity is also the intake of vitamins and supplements to improve sport performances. For instance, athletes often take antioxidants to counteract oxidative stress which occurs during exercise. Among antioxidants, polyphenols have been shown to stimulate the growth of beneficial bacteria such as lactobacilli, bifidobacteria, *Akkermansia*, *Faecalibacterium prausnitzii* and *Roseburia* [54,55,56]. Similarly, branched chain amino acid supplementation to improve muscle building has been shown to produce an enrichment in *Akkermansia* and *Bifidobacterium* in mice [57], whereas other studies have shown that long-term protein supplementation could be harmful for athletes’ microbiota, by producing a decrease in health-promoting bacteria such as *Roseburia*, *Blautia* and *Bifidobacterium longum* [58]. Reduced physical activity during winter could thus partially explain seasonal fluctuations, particularly in populations with sedentary lifestyles.

### 4.5. Circadian Rhythms and Light Exposure Effects on Gut Microbiota

It has been observed that the human circadian rhythms depend also on the seasons [59]. The gut microbiota is tightly synchronized with host circadian rhythms, which themselves are influenced by seasonal changes in daylight duration. Circadian disruption caused by shift work, jet lag or sleep disturbances influence the composition and function of the gut microbiota raising the risk of metabolic diseases such as obesity, insulin resistance, and other NCDs [60]. An important study showed that fecal microbiota transplantation (FMT) from men exposed to jet lag in germ-free mice resulted in obesity and glucose intolerance; this effect was not observed with FMT from men not exposed to jet lag [10]. The circadian rhythm, which regulates the sleep–wake cycle, influences the composition and activity of the microbiota. An animal study by Gao et al. demonstrated that sleep deprivation-induced melatonin reduction was associated with a decrease in anti-inflammatory cytokines, an increase in pro-inflammatory cytokines, colonic mucosal lesions, and reduced diversity and richness of the gut microbiota of mice [61]. Microbial metabolites, particularly SCFAs and bile acids, serve as mediators of microbiota circadian cross-talk, aligning host metabolic activity with environmental cues [62]. The microbiota and circadian rhythm are indirectly impacted by diet and meal timing. While a high-fat diet decreases the number of bacteria that produce SCFAs, a low-fat diet increases their production [63]. It has been demonstrated that these bacterial metabolites affect the expression of specific genes that control the circadian rhythm [63]. During the fasting, or more generally during the active phase of the day, mice microbiota exhibits a significant increase in Firmicutes, while an increase in Bacteroidetes is observed during the resting phase [64]. This is most likely due to the fact that Firmicutes are commonly known to break down sugars from food, while Bacteroidetes specialize in breaking down glycans found in intestinal mucin, a component of the mucus that lines the intestine [65]. Recent studies highlight that even time-of-day sampling can influence microbial profiles, underscoring the importance of temporal standardization in microbiome research [66].

## 5. Implications for Non-Communicable Chronic Diseases

In general, the gut microbiota of healthy people is stable [67], but changes in composition may be observed as the seasons change. On the other hand, individuals with chronic diseases may show more important changes, which may influence the course of the disease. Patients with NCDs frequently have a compromised gut microbiota, which is characterized by low diversity and an imbalance of beneficial and harmful bacteria. Given that gut microbiota composition shifts seasonally, it is plausible that disease symptoms and metabolic parameters fluctuate as well. In individuals with type 2 diabetes (T2D) seasonal changes in insulin sensitivity have been observed and it may be partially mediated by alterations in the gut microbiota, particularly through changes in SCFA production and gut barrier function [68]. A meta-analysis by Moon et al. showed that there is a significant correlation between IBD exacerbations and seasonal fluctuations most evident in the Crohn’s disease subgroup [69]. It was observed that in patients with IBD, the gut microbiota underwent fluctuations during seasonal changes with a greater abundance of oral commensals *Actinomyces* and *TM-3* in autumn and spring and that these bacteria may be correlated with IBD through the production of hydrogen sulfide [69]. The researchers hypothesized that due to their medical condition, patients with IBD and particularly with Crohn’s disease adopt a diet high in carbohydrates and low in fat which could explain the presence of dental caries and thus genera such as *Actinomyces* in addition to the changes in the oral microbiota that occur during the seasons [70]. In gout, a condition marked by episodic inflammatory flares [71], researchers discovered a seasonal pattern of exacerbations, particularly in spring, despite blood uric acid levels rising in the summer. This paradox could be explained by immune responses and changes in the gut microbiome [71]. The abundance of species such as *Bacteroides caccae* and *Bacteroides xylanisolvens* increased during spring flares, suggesting a microbiota-immune axis as a mediator of disease activity beyond metabolic parameters alone [72].

Similar dynamics are observed in pediatric bronchial asthma, whose seasonal recurrences of exacerbations are associated with specific changes in the nasal microbiota, such as increased *Moraxella* and *Haemophilus* in the fall, that amplify the bronchial inflammatory response [73]. These patterns suggest that seasonal microbial shifts, both in the respiratory tract and gut, may serve as early indicators or modulators of disease flares.

## 6. Methodological Strategies for Clinical Trials

**Figure 2:**
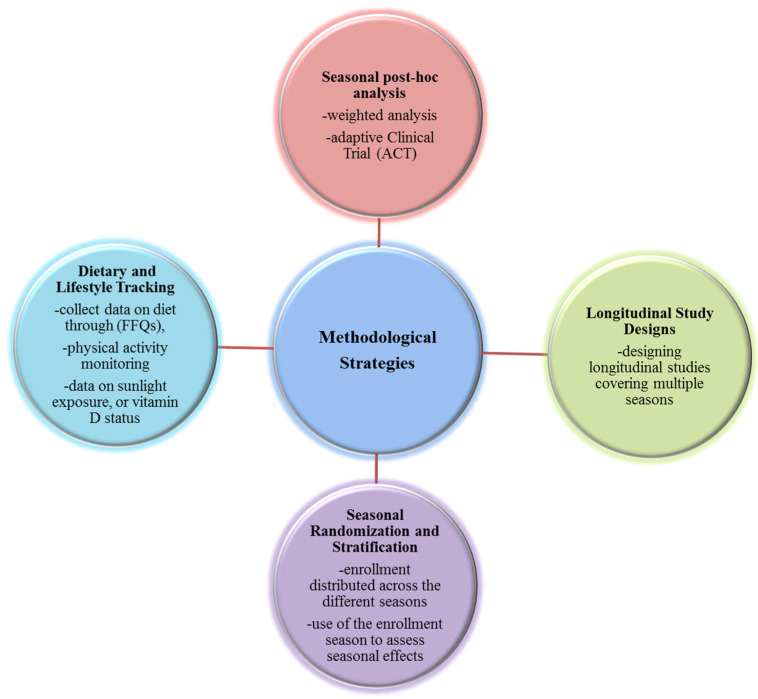
Methodological strategies for Clinical Trials to minimize bias. Diagram summarizing methodological strategies to minimize the confounding effect of seasonality in microbiota-targeted clinical trials. Approaches include longitudinal study design spanning multiple seasons, seasonal stratification during randomization, detailed tracking of seasonal covariates (diet, activity, vitamin D), post hoc statistical corrections such as Inverse Probability Weighting, and use of adaptive clinical trial designs. These methods enhance internal validity by reducing biases introduced by seasonal fluctuations in microbial composition and host physiology.

Seasonality represents a potential confounding factor in the characterization of the gut microbiota. Cross-sectional studies of human populations have demonstrated significant variations in microbial composition between seasons; similarly, animal models have shown seasonal elevations in alpha diversity and changes in major microbial taxa. Such variations, related to environmental, dietary and behavioral fluctuations, may introduce biases into clinical trials of probiotics. To mitigate this methodological risk, preventive measures should be taken, such as stratified randomization by season or parallel enrollment in different arms and adoption of post hoc corrective analytical strategies (Figure 2). A practical example of seasonal microbiota variation is provided by Davenport et al., who conducted a longitudinal study in a population of Hutterites, observing significant compositional shifts between summer and winter using mixed linear models and repeated-measures analysis [7]. Similarly, a large cross-sectional study in the Ukrainian population showed seasonal shifts in major phyla such as Firmicutes and Bacteroidetes, analyzed through Kruskal–Wallis tests and logistic regression models with season as a covariate [13]. These cases illustrate how both design and analysis must account for seasonal patterns to ensure reliable inference.

### 6.1. Longitudinal Study Designs

Rather than conducting short-term studies that capture only one season, researchers should design longitudinal trials that span multiple seasons. This approach allows for the assessment of seasonal variations in gut microbiota composition and probiotic efficacy over time. Statistically, this enables the use of linear mixed-effects models, where the subject is treated as a random effect and the season as a fixed effect. Such models control for intra-individual variability and isolate the seasonal component of change.

### 6.2. Seasonal Randomization and Stratification

To minimize biases, patient recruitment should be evenly distributed across different seasons. Alternatively, stratification based on the season of enrollment could be used to analyze potential seasonal effects on probiotic response. For example, in the case of a hypothetical study on probiotics in which most of the treatment group is enrolled in the summer and the control group in the winter, the microbial differences observed could reflect seasonal variation rather than the effect of the treatment. Including season as a covariate in regression models or performing interaction analyses would help disentangle these effects. In addition, stratified randomization by season ensures balance between arms. Subjects can also be randomized by season (i.e., stratify recruitment so that each arm has the same number of participants per season), or the trial should be designed so that each subject is measured in at least two seasons.

### 6.3. Dietary and Lifestyle Tracking

Since diet and lifestyle factors are major seasonal covariates and influence gut microbiota composition, collecting detailed dietary records and physical activity data throughout the study period would help control for confounding variables during trials. Recommended measures include food frequency questionnaires (FFQs) tailored to local seasonal availability; activity tracking (e.g., step counts, exercise logs); data on sunlight exposure or vitamin D status. Controlling for these variables enhances the validity of microbiota-based inferences and helps isolate the effect of the intervention from seasonal behaviors.

### 6.4. Seasonal Post Hoc Analysis

When prospective control of seasonal distribution is not possible, statistical techniques can help correct for seasonal imbalance. In particular, weighted analysis allows for rebalancing the seasonal distribution between groups by assigning weights to subjects via Inverse Probability Weighting, which assigns each subject a weight inversely proportional to the probability of enrollment in the season in which he or she was included, improving the internal validity of the estimates, especially when balanced enrollment could not be guaranteed. These approaches do not replace a robust design, but are an effective complement for sensitivity analyses and correction of any imbalances [74]. Another approach that allows us to consider the seasonality variable is the use of an Adaptive Clinical Trial (ACT) that can mitigate the bias introduced by unbalanced enrollments by offering greater statistical power by adapting the design to changing biological realities. An adaptive trial is a clinical trial in which certain aspects of the experimental design can be modified as the trial progresses, building on the data accumulated to that point, without compromising statistical integrity [75]. Modifications may concern the following:

- the sample size (sample size re-estimation);
- the randomization (response-adaptive randomization);
- the duration of follow-up;
- even the early closure of an arm.

Seasonality introduces non-negligible biological variability in microbiota data. An adaptive design can monitor and correct for seasonal imbalances. If the trial is conducted along multiple seasons (e.g., enrollment from January to December), an ACT can (i) monitor the seasonal distribution of subjects in the groups, if imbalances emerge (e.g., more treated in the summer); (ii) adjust randomization rates or stratify new enrollments to balance the seasons; e.g., if at mid-study it is noted that the treatment group has an excess of subjects enrolled in summer, randomization can be adjusted to enroll more subjects in the control group during summer; (iii) update treatment effect estimates by correcting for harvest season, in real time, or identify seasonally sensitive sub-populations (e.g., subjects with more unstable gut microbiota in winter). In addition, seasonality can increase intra-subject variability. Adaptive design allows for extending follow-up in subgroups with high variability, or discontinuing early for futility if seasonal fluctuations make it difficult to observe a robust effect. Finally, an ACT allows for adopting flexibility in the timing of enrollment; if a season negatively impacts sample quality (e.g., summer increases fecal sample contamination), an adaptive trial can slow or temporarily suspend enrollment and re-modulate the timing to maximize data quality.

In addition, another tool to correct the bias of seasonality on gut microbiota composition in clinical trials is represented by post hoc analyses such as Tukey’s method, Bonferroni correction or multiple comparisons, which allow for mitigating the systematic variations in data that are tied to different time periods in the enrollment. For instance, Lelwala et al. performed a Kruskal–Wallis test followed by a post hoc multiple comparisons analysis to mitigate the effects of seasonality in tourist arrivals in Sri Lanka [76].

## 7. Conclusions

Scientific evidence accumulated in recent years underscores how seasonality represents a major modulating factor in the composition and function of the gut microbiota. Periodic variations in environmental, dietary, and behavioral elements—such as nutrient availability, sunlight exposure, environmental temperatures, and physical activity levels—substantially influence the gut ecosystem as summarized in Table 1. Moreover, these seasonal fluctuations of gut microbiota could likely worsen the clinical manifestations in people affected by some chronic diseases which are known to be associated with dysbiosis and to be subjected to seasonal exacerbation. While this seasonal plasticity reflects the adaptive capacity of the microbiota, it also introduces a source of variability that is often overlooked in the design and analysis of clinical studies. In particular, the lack of temporal standardization in sample collection can generate methodological biases that compromise data comparability and reproducibility, hindering the identification of reliable correlations between gut microbiota composition and chronic non-communicable diseases (NCDs). To improve the accuracy, reliability, and clinical relevance of microbiota-based probiotic studies, researchers should incorporate seasonality into their study designs through longitudinal approaches, balanced recruitment, and diet and lifestyle assessments. If probiotic treatments aim to restore microbial balance, their efficacy may be influenced by the timing of administration. Given the seasonal fluctuation in baseline gut microbiota composition, identical probiotic interventions could yield variable outcomes depending on the time of year. This has significant implications for clinical trial design and therapeutic application, such as ignoring seasonality risks, underestimating or overestimating probiotic effects or misattributing observed changes to the intervention when they may reflect natural seasonal variation. In addition to improving the quality of the studies, such an approach may foster a more precise understanding of the mechanisms by which the gut microbiota contributes to the pathogenesis of NCDs offering new perspectives for prevention and personalized treatment. In conclusion, the seasonality of the gut microbiota is still underexplored in clinical settings, deserves more attention as a biologically significant variable. Its systematic recognition may not only strengthen the robustness of microbiome studies but also pave the way for more targeted, flexible and time-sensitive therapeutic interventions in line with the principles of precision medicine. However, evidence from human population studies exists but is limited, and more longitudinal work on various populations is still needed to confirm the impact of seasonal variations on microbiota.

## Figures and Tables

**Table 1:** Environment, diet, sunlight exposure, temperatures, circadian rhythms and physical activity depend on seasonal variations and in turn influence the gut microbiota.

**Seasonal Factor**	**Population**	**Method**	**Gut Bacteria Over-Represented**	**Gut-Bacteria Under-Represented**	**Ref.**
High-fat, high-protein diets	Hutterites	16S rRNA gene sequencing	Actinobacteria	Bacteroidetes	[7]
High fiber and low protein diets	Hutterites	16S rRNA gene sequencing	Bacteroidetes	Actinobacteria, Firmicutes	[7]
Sunlight and vitamin D	Slovaks	16S rRNA gene sequencing 16S rRNA gene sequencing	Enterobacteriaceae, *Roseburia*,	*Eggerthella lenta*, *Helicobacter*,	[21,23]
	German		Alistipes, Faecalibacterium	*Fusobacterium*, *Faecalibacterium prausnitzii*	
Physical Activity	Spanish	16S rRNA gene sequencing	*Faecalibacterium prausnitzii*, *Roseburia hominis*, *Akkermansia muciniphila*	_	[50]
	Japanese	16S rRNA gene sequencing	*Faecalibacterium*, *Streptococcus*		[51]
Circadian Rhythms, active phase	murine models	16S rRNA gene sequencing	Firmicutes	Bacteroidetes	[64]
Circadian Rhythms, resting phase	murine models	16S rRNA gene sequencing	Bacteroidetes	Firmicutes	[64]
Heat Temperature	Chinese	16S rRNA gene sequencing	*Escherichia*, *Shigella*	*Dorea*, *Blautia*, *Lactobacillus*, *Subdoligranulum*	[33]
Cold Temperature	murine models	16S rRNA gene sequencing	Firmicutes/Bacteroidetes ratio	*Akkermansia muciniphila*	[35]
Gout exacerbation in spring	Chinese	16S rRNA gene sequencing	*Bacteroides caccae*, *Bacteroides xylanisolvens*	SCFA-producing bacteria	[72]
IBD in summer/autumn	Slovaks	16S rRNA gene sequencing	*Pediococcus*, *Clostridium*, *Escherichia/Shigella*	*Eggerthella lenta*, *Helicobacter*, *Fusobacterium*, *Faecalibacterium prausnitzii*	[21]
Respiratory tract infections	Chinese	16S rRNA gene sequencing	Enterobacteriaceae	*Lactobacillus*	[41]
	Chinese	16S rRNA gene sequencing	*Enterococcus*	*Bifidobacterium*	[42]

## Data Availability

No new data were created or analyzed in this study. Data sharing is not applicable to this article.
